# Supplementary material for: Unraveling exciton–phonon coupling in individual FAPbI3 nanocrystals emitting near-infrared single photons
Source: Nat Commun. 2018 Aug 20;9:3318. doi: 10.1038/s41467-018-05876-0 (PMC6102301; doi:10.1038/s41467-018-05876-0)
Supplement: Supplementary file 1 — Supplementary Information [file 41467_2018_5876_MOESM1_ESM.pdf]

Supplementary Information

Unraveling exciton-phonon coupling in individual $FAPbI_3$ nanocrystals emitting near-infrared single photons

Ming Fu,[†,‡] Philippe Tamarat,[†,‡] Jean-Baptiste Trebbia,[†,‡] Maryna I. Bodnarchuk,[¥] Maksym V. Kovalenko,[¥,§] Jacky Even,[¶] and Brahim Lounis*[†,‡]

[†] *Université de Bordeaux, LP2N, Talence F-33405, France,*

[‡] *Institut d'Optique and CNRS, LP2N, Talence F-33405, France,*

[¶] *Univ Rennes, INSA Rennes, CNRS, Institut FOTON - UMR 6082, F-35000 Rennes, France*

[¥] *Laboratory for Thin Films and Photovoltaics, Empa-Swiss Federal Laboratories for Materials Science and Technology, CH-8600 Dübendorf, Switzerland*

[§] *Institute of Inorganic Chemistry, Department of Chemistry and Applied Biosciences, ETH Zürich, CH-8093 Zürich, Switzerland*

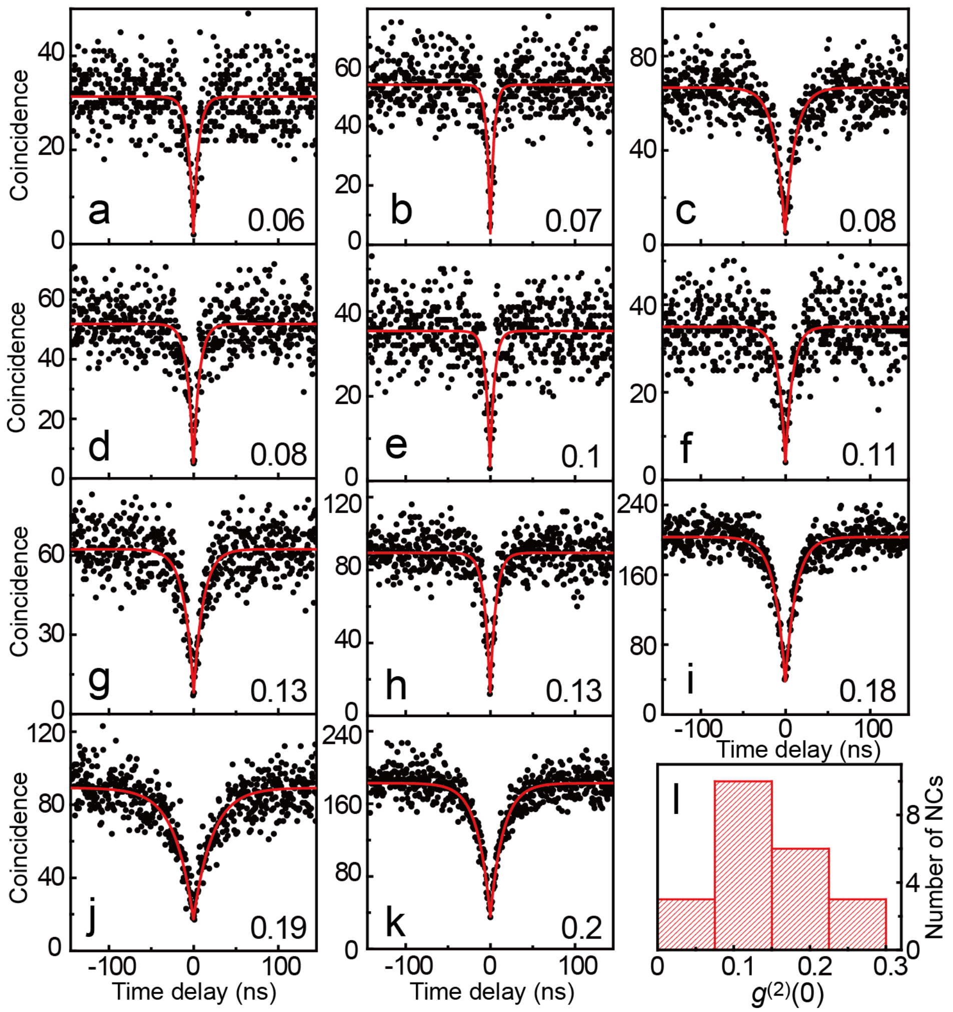

Supplementary Figure 1: Fluorescence intensity autocorrelation function of several $FAPbI_3$ single NCs at room temperature.

(**a-k**) Histograms of time delays between consecutive photon pairs from the PL of individual $FAPbI_3$ NCs. (**l**) Distribution of $g^{(2)}(0)$ values. The main contribution to $g^{(2)}(0)$ is attributed to residual biexciton radiative recombination.

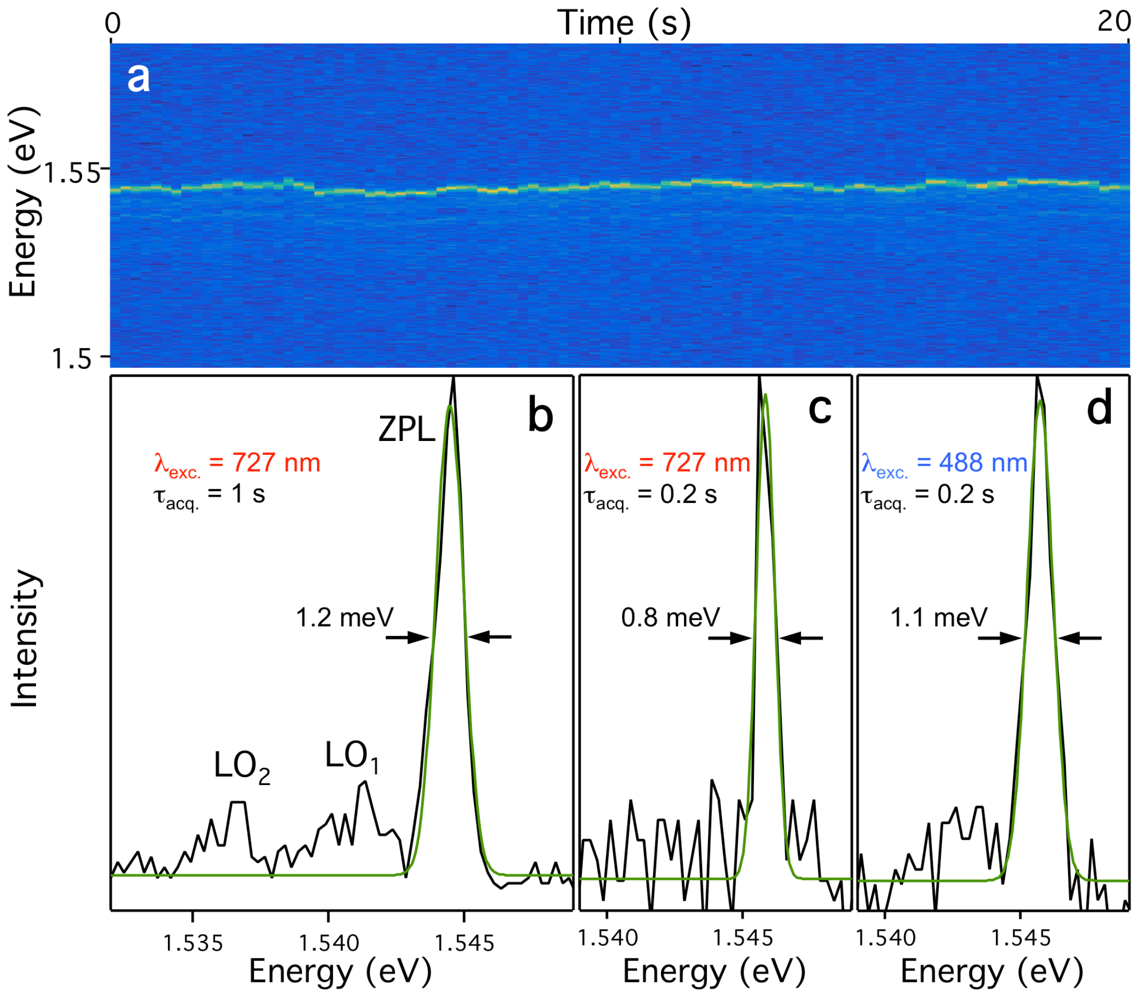

Supplementary Figure 2: **PL spectrum of a single $FAPbI_3$ NC at 3.6 K.**

This NC is the same as that of Fig. 2 in the main text. (**a**) Spectral trajectory of the emission line of a single NC at 3.6 K. Each spectrum of the trajectory was recorded over 0.2 s with an excitation wavelength of 727 nm. (**b**), (**c**), (**d**) PL spectra of the same NC recorded over 1s (**b**), 0.2 s at an excitation wavelength of 727 nm (**c**), and over 0.2 s with an excitation wavelength of 488 nm (**d**). Fitting with Gaussian profiles (green curves) yields FWHM linewidths of 1.2 meV (**b**), 0.8 meV (**c**) and 1.1 meV (**d**) for the ZPL.

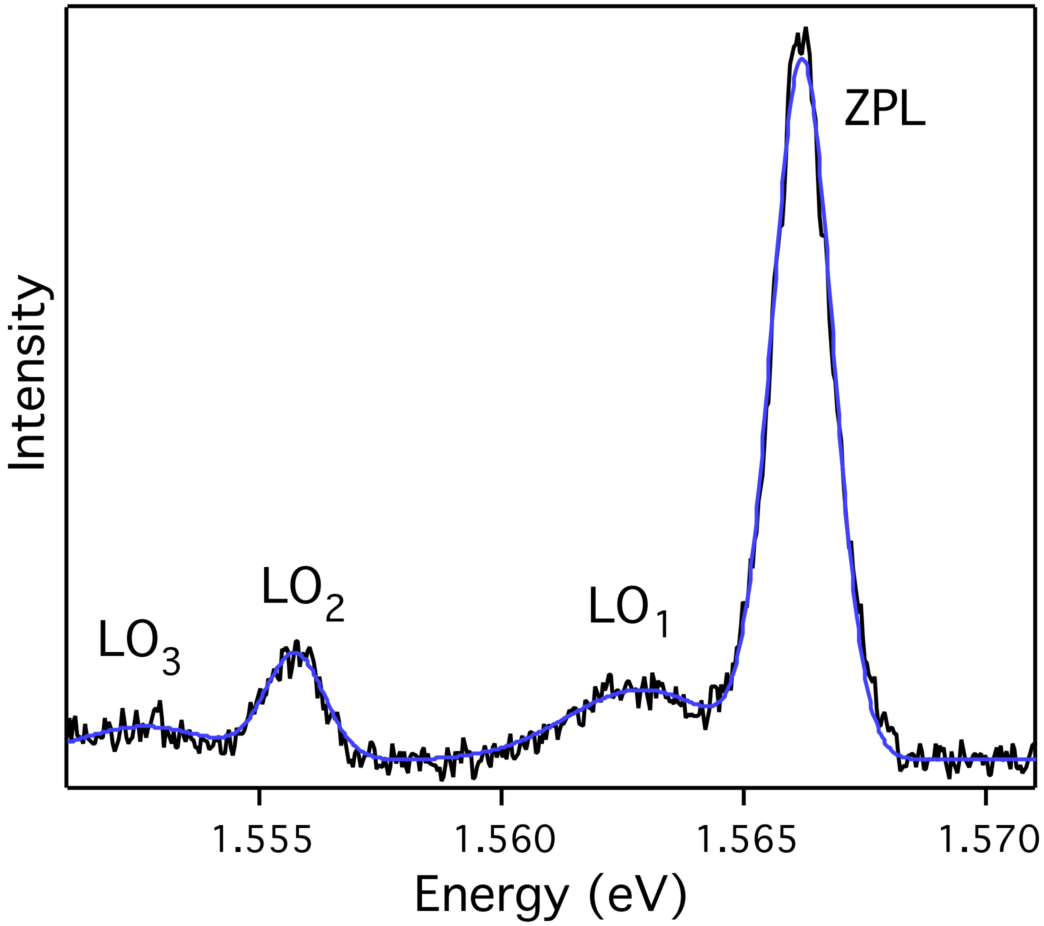

Supplementary Figure 3: **High-resolution PL spectrum of a single $FAPbI_3$ nanocrystal at 3.6 K.**

The spectrum is recorded over an integration time of 0.5 s, with a grating of 1800 lines/mm giving a spectrograph resolution of ~120 μeV. Fitting with a triple-Gaussian profile (blue curve) gives the LO-phonon energies E_{LO1} = 3.4 meV, E_{LO2} = 10.6 meV, E_{LO3} = 13.6 meV, and the FWHM linewidths 1.5 meV, 3.6 meV, 1.4 meV and 3.3 meV for the ZPL, LO_1, LO_2 and LO_3 sidebands, respectively.

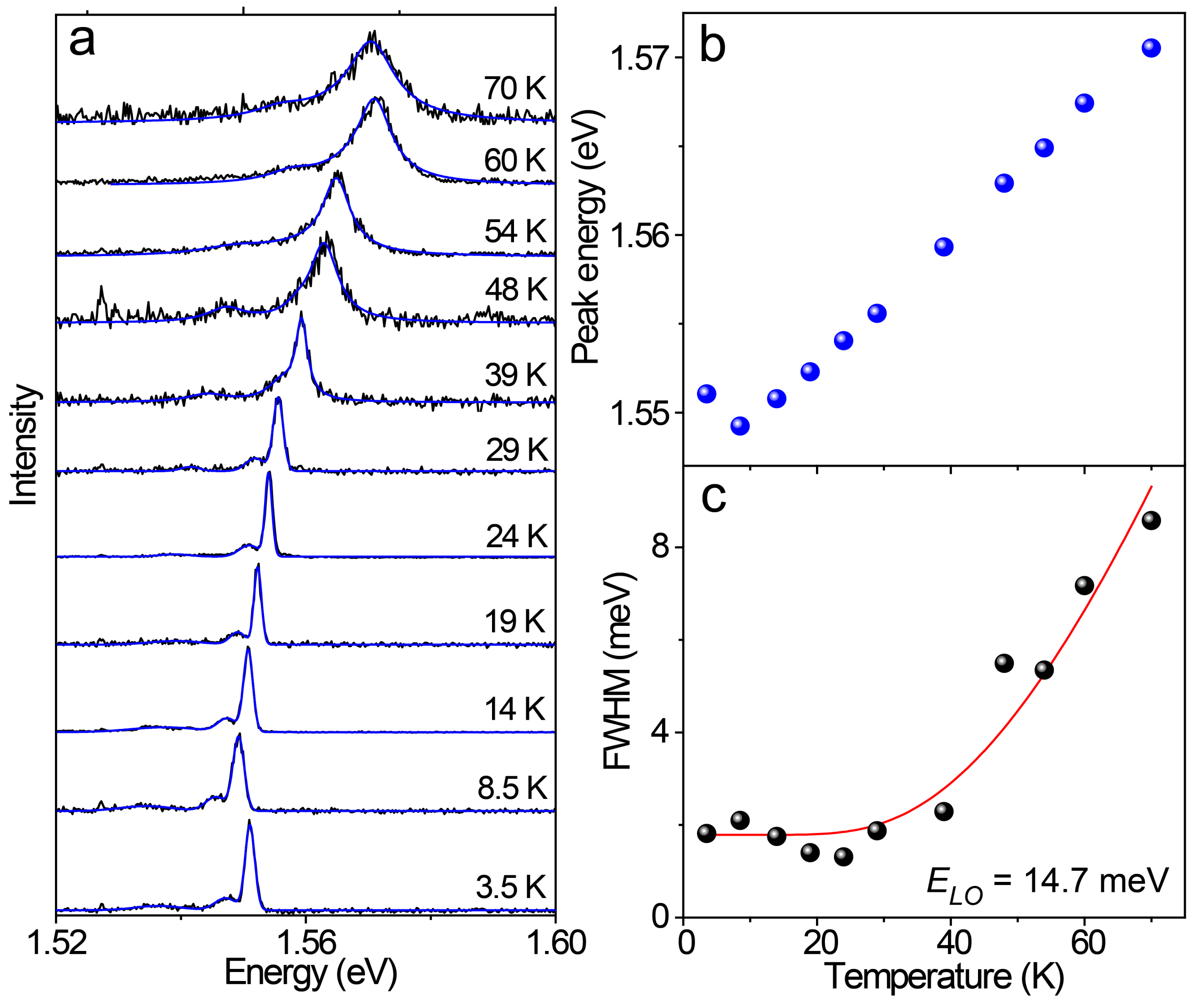

Supplementary Figure 4: Temperature dependence of the PL spectrum of a $FAPbI_3$ nanocrystal.

The evolution with temperature of the FWHM linewidth is fitted, Γ_0 = 1.5 meV, Γ_{LO} = 38.4 meV, and E_{LO} = 10.4 meV.

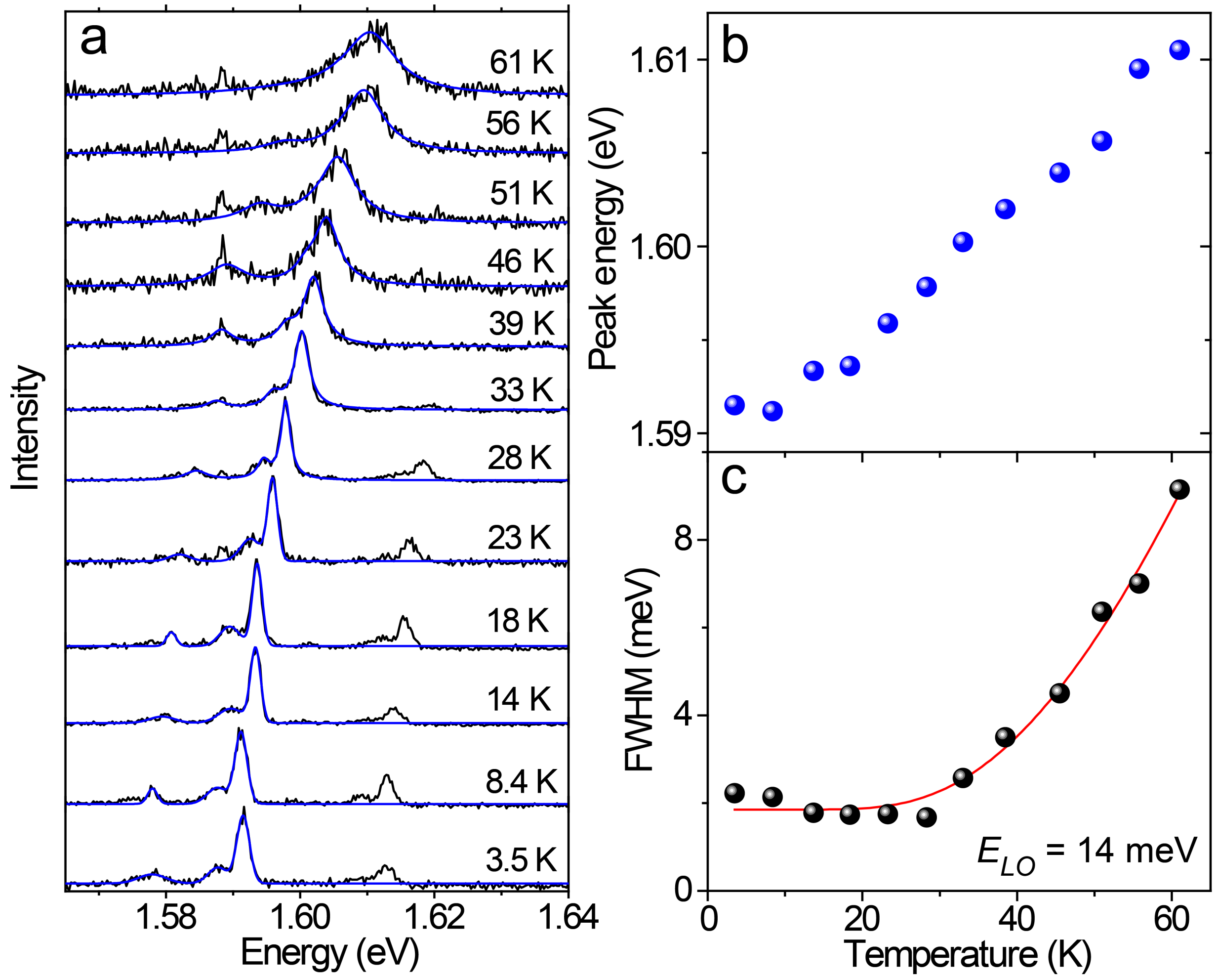

Supplementary Figure 5: Temperature dependence of the PL spectrum of a $FAPbI_3$ nanocrystal.

The evolution with temperature of the FWHM linewidth is fitted, $\Gamma_0 = 1.8$ meV, $\Gamma_{LO} = 62.3$ meV, and $E_{LO} = 11.6$ meV.

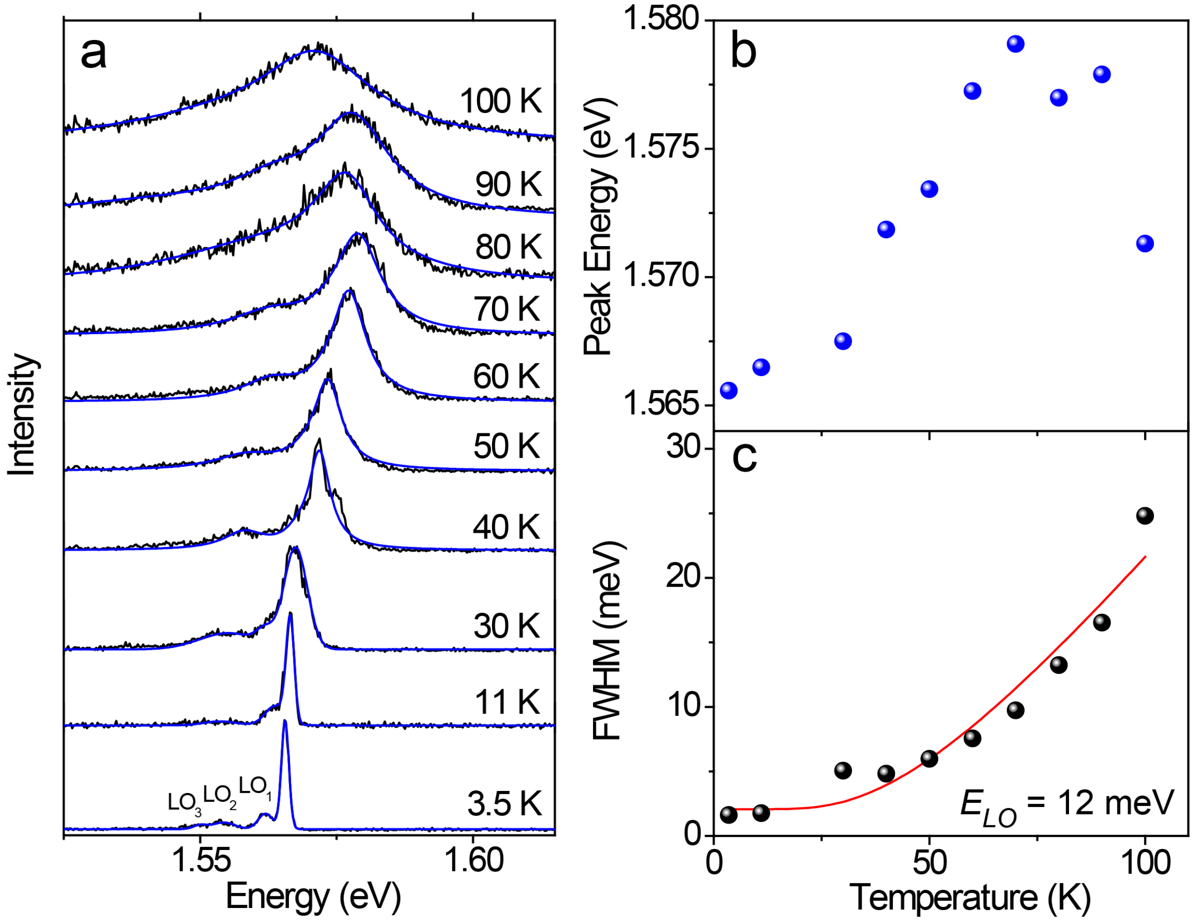

Supplementary Figure 6: Temperature dependence of the PL spectrum of a $FAPbI_3$ nanocrystal.

The evolution with temperature of the FWHM linewidth is fitted, Γ_0 = 1.6 meV, Γ_{LO} = 71.8 meV, and E_{LO} = 12 meV.

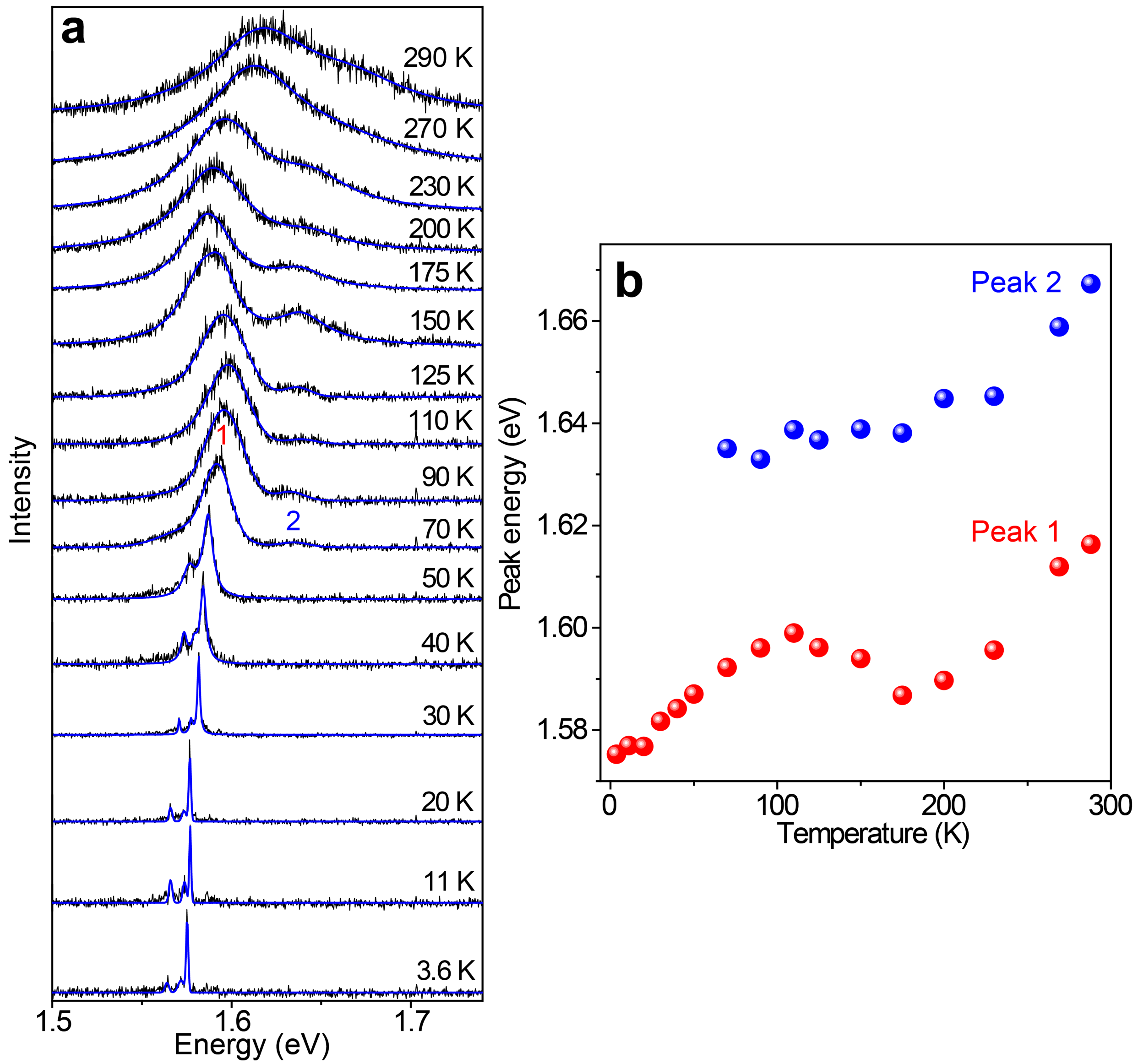

Supplementary Figure 7. Temperature dependence of the PL spectrum of a $FAPbI_3$ nanocrystal.

For this NC, the turning point in the PL spectrum evolution goes with the onset of a new emission line at higher energy, which remains up to room temperature. This behavior might be due to the coexistence of two phases over a wide range of temperatures. Less than 10% of the nanocrystals display a similar behavior.

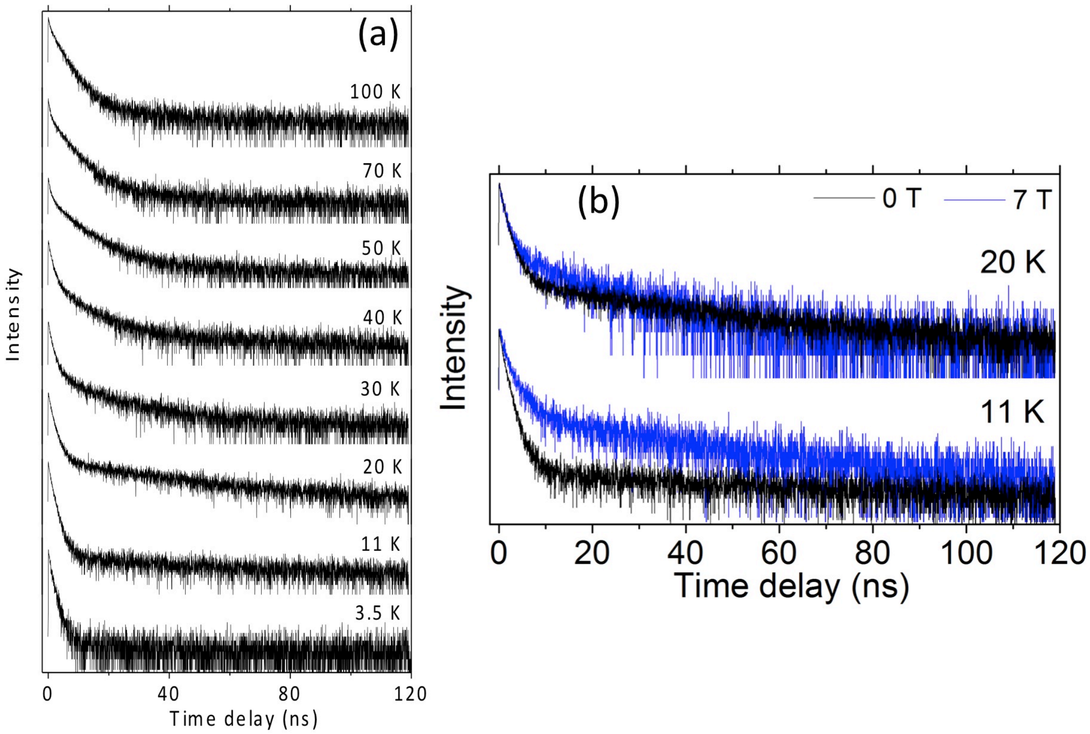

Supplementary Figure 8. Magnetic field effect on the PL decay.

(**a**) Temperature evolution of the PL decay for a single FAPbI3 NC. (**b**) Under a magnetic field the long time component shortens and gains weight. The magnetic brightening of the dark state is less pronounced at 20 K than at 11 K due to a stronger thermal mixing between the bright and dark states.

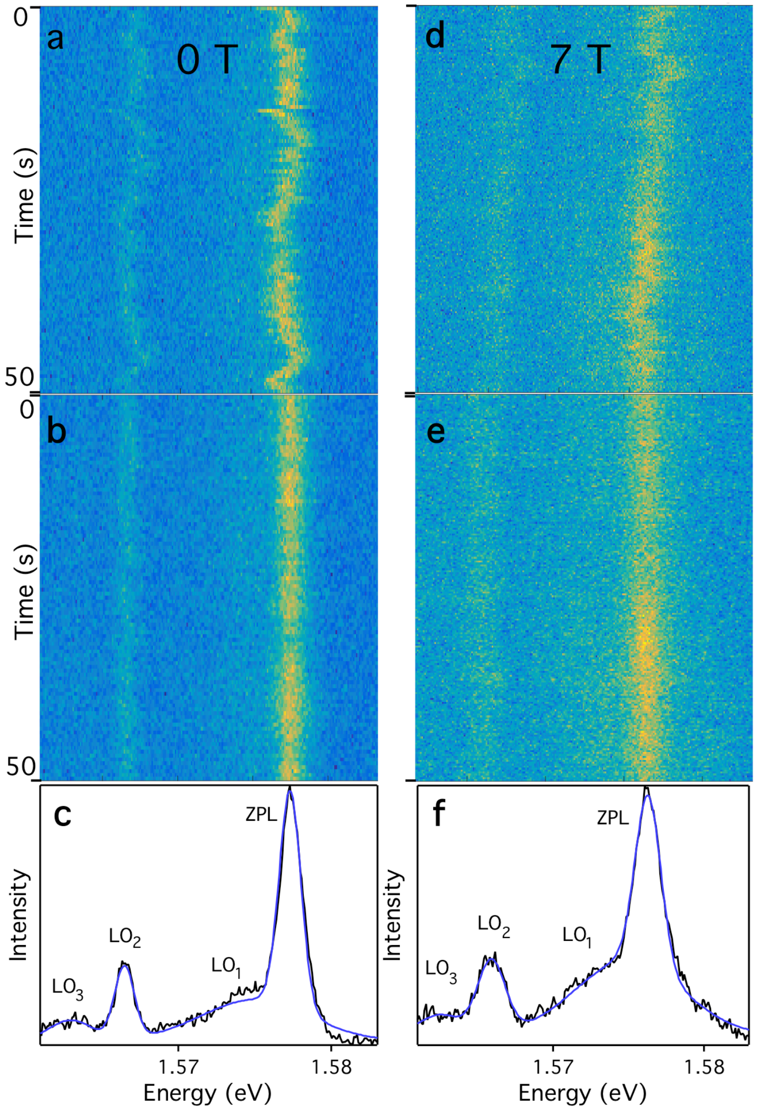

Supplementary Figure 9. PL spectra of a single $FAPbI_3$ nanocrystal at 0 T and at 7 T.

Spectral trajectories of a single nanocrystal recorded at 0 T (**a**) and 7 T (**d**) at 3.6 K. The integration time of each spectrum is 0.5 s. After centering the PL spectra to eliminate the ZPL spectral diffusion, we obtain the trails (**b**) at 0 T and (**e**) at 7 T. The sum of all centered spectra of the trails (**b**) and (**e**) are displayed in (**c**) and (**f**), respectively. Fitting with a quadruple-Gaussian profile (blue curves) gives the FWHM ZPL-linewidths of 1.8 meV and 2.0 meV at 0 T and 7 T, respectively.

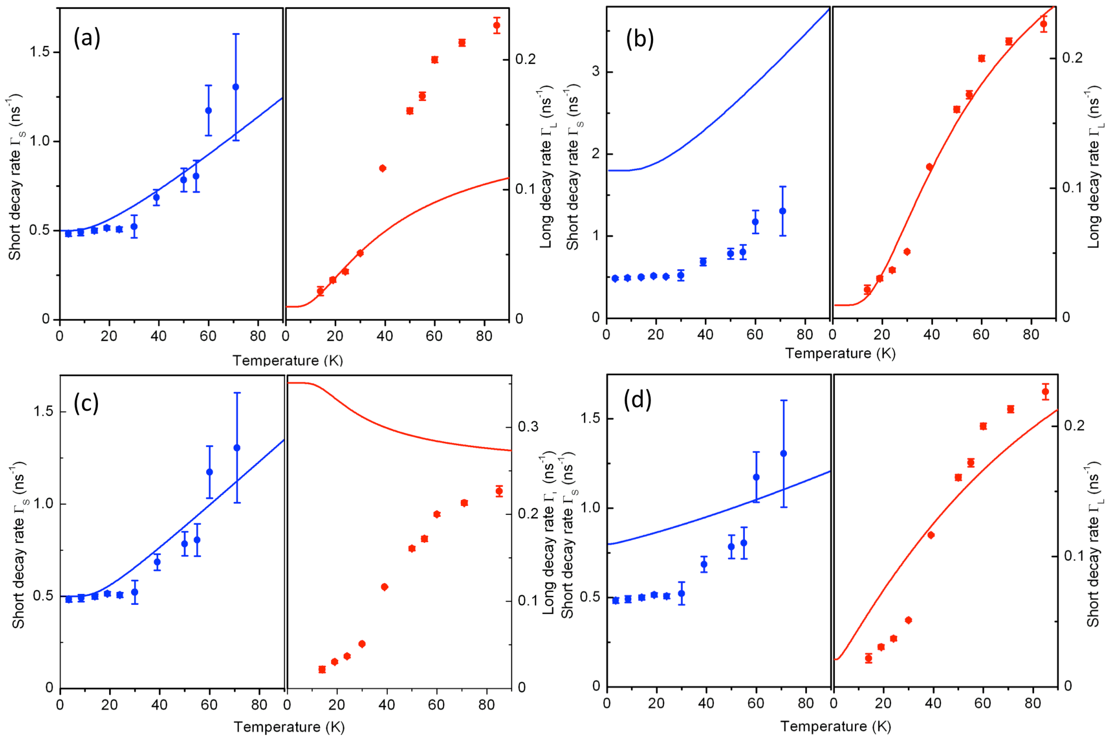

Supplementary Figure 10. Attempts to reproduce the temperature dependence of the PL decay with the one-phonon thermal mixing model.

In this model, emission and absorption of acoustic phonons from a mode whose energy E_{Ac} matches the bright-dark splitting induces a thermalization of the excitonic states. The transition rates between bright and dark levels are $\gamma_{\uparrow} = \gamma_0^{Ac} N_B$ and $\gamma_{\downarrow} = \gamma_0^{Ac}(N_B + 1)$, where $N_B = 1/[\exp(E_{Ac}/k_B T) - 1]$ is the Bose-Einstein phonon number at the temperature T. The decay rates of the short (blue points) and the long (red points) components, together with their error bars, are the same as in Fig. 4. In the simulations (solid curves), the dark level is placed below (**a** and **b**) or above (**c** and **d**) the bright level. The temperature dependence of the short and the long decay components cannot be reproduced with the same set of parameters, whatever the bright-dark level ordering. (**a**) The evolution of the short component is reproduced with the parameters Γ_B= 0.3 ns^{-1}, Γ_D=0.01 ns^{-1}, γ_0^{Ac}=0.2 ns^{-1}, E_{Ac}= 3 meV. (**b**) The evolution of long component is reproduced with the parameters Γ_B= 0.8 ns^{-1}, Γ_D=0.01 ns^{-1}, γ_0^{Ac}=1 ns^{-1}, E_{Ac}= 5 meV. (**c**) The evolution of the short component is reproduced with the parameters Γ_B= 0.5 ns^{-1}, Γ_D=0.001 ns^{-1}, γ_0^{Ac}=0.35 ns^{-1}, E_{Ac}= 5 meV. (**d**) No set of parameters can be found to reproduce the evolution of the long component. This evolution is very roughly approached with the parameters Γ_B= 0.8 ns^{-1}, Γ_D=0.001 ns^{-1}, γ_0^{Ac}=0.02 ns^{-1}, E_{Ac}= 0.5 meV.

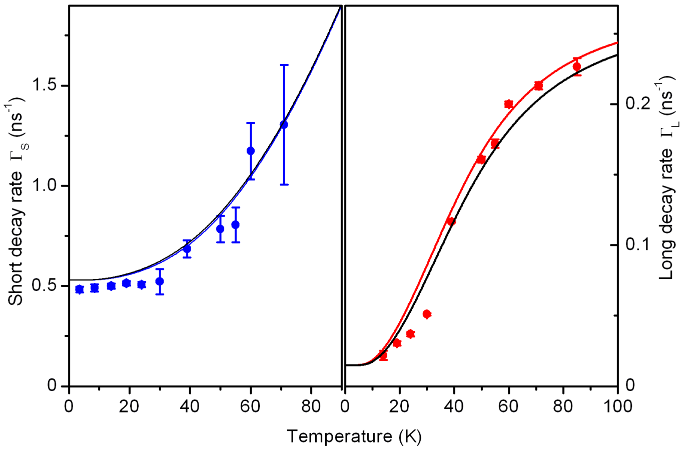

Supplementary Figure 11. Modeling the temperature dependence of the PL decay with the two-phonon thermal mixing model, taking the dark level below or above the bright one.

The decay rates of the short (blue points) and the long (red points) components, together with their error bars, are the same as in Fig. 4. The blue curve and the red curve are the same simulated evolutions as in Fig. 4c and d. They are obtained by taking the dark level below the bright, and the parameters Γ_B= 0.53 ns^{-1}, Γ_D=0.015 ns^{-1}, γ_0=0.16 ns^{-1}, E_{LO1A}= 3.3 meV, E_{LO1B}= 3.6 meV. For comparison, the black curves are the simulated evolutions obtained after reversing the order of bright and dark levels in the model.

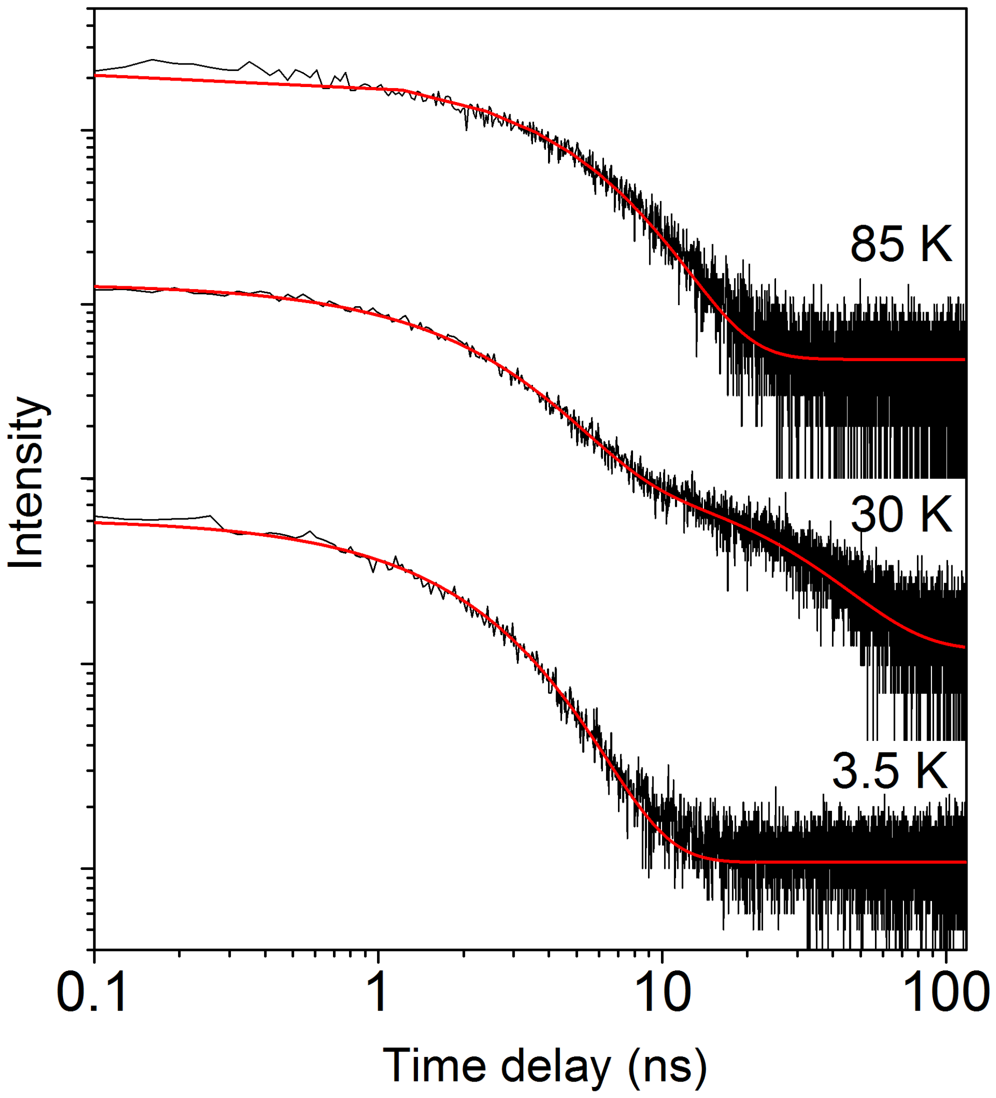

Supplementary Figure 12. Temperature dependence of the PL decay of a single $FAPbI_3$ NC in log-log scale.

The PL decay and fitting curves (red curves) are the same as those presented in Fig. 4a.

Supplementary Note 1: Two LO-phonon relaxation process in a halide perovskite nanocrystal

Usually, for small energy splittings between fine structure sublevels, the interactions with acoustic phonons are the dominant relaxation processes. However, in these soft materials displaying a high level of disorder in the FA cation positions, the interaction of the excitons with acoustic phonons is drastically reduced. In contrast, quasi-elastic two-phonon relaxation processes, in which one LO phonon is absorbed and another is emitted, can lead to efficient interlevel thermalization. The temperature dependence of the experimental decay rates is well reproduced using a two LO-phonon process. This observation is consistent with a dominant Fröhlich interaction associated with low energy optical phonons.

Relaxation rates associated to two-phonon processes may be computed from the second order Fermi golden rule [1,2]:

$$1/\tau = \frac{2\pi}{\hbar}\sum_{\boldsymbol{q}}\sum_{\boldsymbol{k}}\left|\sum_s' \frac{M_{\boldsymbol{q}}^{\mathrm{is}}M_{\boldsymbol{k}}^{\mathrm{sf}}}{E_{\mathrm{i}}-E_s+\hbar\omega_{\boldsymbol{q}}+i\hbar\Gamma_s/2}+\frac{M_{\boldsymbol{k}}^{\mathrm{is}}M_{\boldsymbol{q}}^{\mathrm{sf}}}{E_{\mathrm{i}}-E_s-\hbar\omega_{\boldsymbol{k}}+i\hbar\Gamma_s/2}\right|^2 N_{\boldsymbol{q}}(N_{\boldsymbol{k}}+1)\delta\left(E_{\mathrm{f}}-E_{\mathrm{i}}-\hbar\omega_{\boldsymbol{q}}+\hbar\omega_{\boldsymbol{k}}\right)$$
(1)

where $N_{q(k)} = 1/\left[\exp\left(\hbar\omega_{\boldsymbol{q}(\boldsymbol{k})}/k_{\mathrm{B}}T\right)-1\right]$ is the Bose-Einstein occupation number for the absorbed (emitted) phonon, $1/\Gamma_s$ is the lifetime of the intermediate state s, $M_{\boldsymbol{q}(\boldsymbol{k})}^{ab}$ are the matrix elements, $E_{\mathrm{i},s,\mathrm{f}}$ are the energy levels of the initial, intermediate and final states.

Matrix elements for the Fröhlich interaction with optical phonons $\hbar\omega_{\boldsymbol{q}}$ are :

$$M_{\boldsymbol{q}}^{ab} = i\sqrt{\frac{e^2\hbar\omega_{\boldsymbol{q}}}{2q^2V\varepsilon_0}\left(\frac{1}{\varepsilon_\infty^r}-\frac{1}{\varepsilon_s^r}\right)}\ \left\langle\Psi_a^{e(h)}(\boldsymbol{r})\middle|e^{i\boldsymbol{q}.\boldsymbol{r}}\middle|\Psi_b^{e(h)}(\boldsymbol{r})\right\rangle \quad (2)$$

where $\varepsilon_{\infty(s)}^r$ is the high frequency(static) dielectric constant and $\Psi_a^{e(h)}(\boldsymbol{r})$ and $\Psi_b^{e(h)}(\boldsymbol{r})$ are the electron(hole) wavefunctions in the initial, intermediate or final states, respectively.

Since the bare electron/hole phonon interaction does not contain spin operators, the matrix elements of the Fröhlich interaction are non-zero only if spin mixing of the involved exciton states occurs. Spin mixing might be related either to the exchange interaction[3] or to the Rashba effect that is in principle triggered by a loss of inversion symmetry and enhanced by the large spin-orbit coupling in Pb-based compounds [4]. Non-centrosymmetry was indeed reported in the early study of the low temperature crystallographic structure of $FAPbI_3$ [5] and was confirmed recently[6]. In order to estimate the characteristic two-phonon mixing rate γ_0, an elaborate description of the electronic structure of these nanocrystals requires detailed information on their low-temperature crystallographic structures, spectroscopic properties, morphology and ligands binding at the surface. This is beyond the scope of the present work.

1. Tsitsishvili, E., Baltz, R. V. & Kalt, H. Temperature dependence of polarization relaxation in semiconductor quantum dots. *Phys. Rev. B* **66,** 87 (2002).
2. Tsitsishvili, E. Light-hole exciton spin relaxation in quantum dots. *Phys. Rev. B* **91,** 178 (2015).
3. Nahálková, P. *et al.* Two-phonon assisted exciton spin relaxation due to exchange interaction in spherical quantum dots. *Phys. Rev. B* **75,** 1031 (2007).
4. Becker, M. A. *et al.* Bright triplet excitons in caesium lead halide perovskites. *Nature* **553,** 189–193 (2018).
5. Stoumpos, C. C. *et al.* Crystal Growth of the Perovskite Semiconductor $CsPbBr_3$: A New Material for High-Energy Radiation Detection. *Cryst. Growth Des.* **13,** 2722–2727 (2013).
6. Weber, O. J. *et al.* Phase Behavior and Polymorphism of Formamidinium Lead Iodide. *Chemistry of Materials* **30,** 3768–3778 (2018).
